# Emerging Role of Long Non-Coding RNAs in Diabetic Vascular Complications

**DOI:** 10.3389/fendo.2021.665811

**Published:** 2021-06-21

**Authors:** Vinay Singh Tanwar, Marpadga A. Reddy, Rama Natarajan

**Affiliations:** Department of Diabetes Complications and Metabolism, Arthur Riggs Diabetes and Metabolism Research Institute, Beckman Research Institute of City of Hope, Duarte, CA, United States

**Keywords:** diabetes, diabetes complications, long non-coding RNAs, inflammation, fibrosis, epigenetics

## Abstract

Chronic metabolic disorders such as obesity and diabetes are associated with accelerated rates of macrovascular and microvascular complications, which are leading causes of morbidity and mortality worldwide. Further understanding of the underlying molecular mechanisms can aid in the development of novel drug targets and therapies to manage these disorders more effectively. Long non-coding RNAs (lncRNAs) that do not have protein-coding potential are expressed in a tissue- and species-specific manner and regulate diverse biological processes. LncRNAs regulate gene expression in *cis* or in *trans* through various mechanisms, including interaction with chromatin-modifying proteins and other regulatory proteins and *via* posttranscriptional mechanisms, including acting as microRNA sponges or as host genes of microRNAs. Emerging evidence suggests that major pathological factors associated with diabetes such as high glucose, free fatty acids, proinflammatory cytokines, and growth factors can dysregulate lncRNAs in inflammatory, cardiac, vascular, and renal cells leading to altered expression of key inflammatory genes and fibrotic genes associated with diabetic vascular complications. Here we review recent reports on lncRNA characterization, functions, and mechanisms of action in diabetic vascular complications and translational approaches to target them. These advances can provide new insights into the lncRNA-dependent actions and mechanisms underlying diabetic vascular complications and uncover novel lncRNA-based biomarkers and therapies to reduce disease burden and mortality.

## Introduction

Diabetes is a global epidemic affecting nearly 463 million people worldwide and is projected to increase by 700 million in 2045 (1). The increasing prevalence of diabetes significantly augments the risk for multiple associated complications, including cardiovascular diseases (such as atherosclerosis and hypertension), diabetic nephropathy (DN; also known as diabetic kidney disease), diabetic cardiomyopathy (DCM), and diabetic retinopathy (DR) (2–4). The morbidity and mortality associated with these complications significantly increase the global economic burden. Therapeutic interventions and lifestyle modifications are helpful to decrease the incidence of diabetes but are not always effective in preventing the progression of vascular complications. Therefore, identification of new mechanisms and related novel therapies to mitigate the impact of diabetes and associated complications is urgently warranted. Chronic high glucose (HG) and increased levels of other pathological factors like advanced glycation end products (AGE)s, saturated free fatty acids (FFAs), and low density lipoprotein (LDL) cholesterol can dysregulate the functions of numerous cell types, including inflammatory and immune cells, endothelial cells (ECs), vascular smooth muscle cells (VSMCs), cardiac myocytes, and renal cells in target tissues, leading to various complications of diabetes (2). Multiple underlying mechanisms such as activation of various signaling pathways and downstream transcription factors that promote expression and production of inflammatory cytokines, fibrotic factors, oxidative stress, and other related changes have been described (2, 5–8). Despite such knowledge of traditional regulatory mechanisms driving cellular dysfunction in diabetic complications, the continued increase in disease incidence underscores the need to explore other mediators and mechanisms. Clinical studies with diabetic patients have revealed that memory of prior episodes of hyperglycemia can have long-term deleterious effects with continued risk and progression of complications despite subsequent glycemic control, a phenomenon known as “metabolic memory” or “legacy effect” (5, 9). In addition, accumulating evidence suggests key roles for epigenetic changes and non-coding RNAs in regulating genes, cellular functions, and phenotypes associated with diabetes complications and metabolic memory. Here we review recent reports on the characterization, functions, and mechanisms of action of long non-coding RNAs (lncRNAs) in diabetic vascular complications and translational approaches to target them.

## Epigenetics and Long Non-Coding RNAs

Genome-wide association studies (GWAS) have implicated multiple risk loci for diabetes complications such as DKD, but the functional significance of many of these genomic regions is not yet fully understood (10). Because many human disease risk loci are often located in non-coding regions and enhancers, they likely influence the expression of associated genes *via* epigenetic mechanisms. Epigenetics plays an important role in the integration of cellular responses to diverse genetic, metabolic, and environmental factors (11, 12) and refers to changes in gene expression and function without alteration in the DNA sequence leading to altered phenotypes (11). Epigenetic modifications, such as chromatin histone post-translational modifications (PTMs) and DNA methylation (DNAme), regulate chromatin structure and function, which are further modulated by non-coding RNAs, such as microRNAs (miRNAs) and lncRNAs. Epigenetic modifications associated with cis-elements, such as proximal promoters and distal enhancers, fine-tune transcriptional activity *via* regulation of chromatin structure and access to transcription factors (TFs). In general, histone PTMs such as histone H3/H4 lysine acetylation (H3/H4K9/14ac) and H3K4methylation (H3K4me1/2/3) regulate transcriptional activation, whereas H3K9me2/3, H3K27me3, and promoter DNAme are associated with gene repression (13). Epigenetic mechanisms are tightly regulated by the balance between the actions of histone or DNA modifying enzymes (“writers”) and actions of enzymes that remove these modifications (“erasers”), and the “readers” that interact with these modifications to regulate chromatin structure (14, 15). LncRNAs can fine-tune these epigenetic processes *via* direct interaction of lncRNAs with chromatin, chromatin modifiers, or other nuclear proteins (16, 17). For instance, the interaction of lncRNA *Xist* with polycomb repressor complex 2 (PRC2), which regulates histone H3K27me3, plays a key role in X chromosome inactivation (17). Whereas interaction of lncRNA *Kcnq1ot1* with the histone H3K9-methyltransferase G9a (EHMT2) increases repressive modification H3K9me2 and inhibits target genes (17). Increasing evidence also implicates the dysregulation of such epigenetic mechanisms and lncRNAs in diabetes and complications (9, 18–21). Further in-depth investigations into the precise mechanisms by which lncRNAs and associated epigenetic mechanisms regulate genes and processes associated with diabetic complications can lead to the development of more effective therapies and clinical management.

## LncRNA-Dependent Mechanisms of Gene Regulation

**Figure 1:**
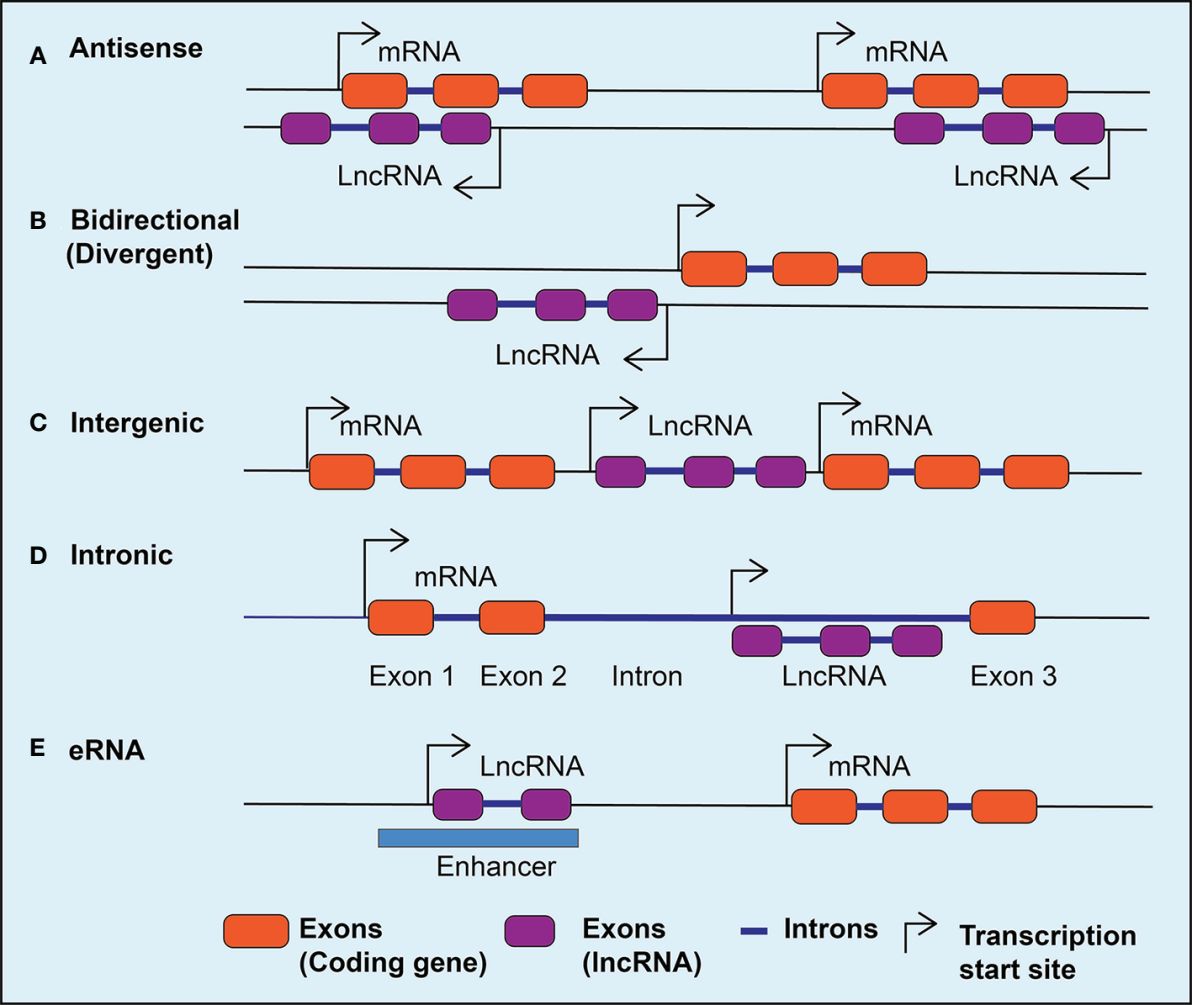
LncRNA classification. Schematic diagram depicting different classes of lncRNAs expressed in mammalian cells. The lncRNAs can be expressed as anti-sense lncRNAs from the anti-sense strand overlapping coding genes **(A)**, as bidirectional or divergent transcripts from a shared promoter with coding genes **(B)**, as intergenic **(C)**, or intronic **(D)**, and as enhancer RNAs (eRNAs) from the enhancers **(E)**. Arrows indicate the direction of transcription.

Advances in the next generation RNA sequencing revealed the complexity and diversity of the transcription process and led to the discovery of thousands of lncRNAs in the mammalian genome (22–24). LncRNAs are transcripts that are >200 nucleotides long and have many features similar to protein-coding genes, including splicing of the transcripts and RNA polymerase II-mediated transcription. However, lncRNAs do not code for any proteins, with the exception of some that are reported to encode small peptides (25). Therefore, while characterizing novel lncRNA transcripts, it is important to use rigorous bioinformatics tools and experimental approaches to verify that they truly lack coding potential (26–29). LncRNAs have also been identified by integrating RNA-seq data with chromatin immunoprecipitation linked to sequencing (ChIP-seq) data, which can reveal enrichment of H3K4me3 marks at transcriptional start sites and H3K36me3 at gene bodies and can distinguish genomic regions from which lncRNAs can be transcribed (30, 31). LncRNAs can be classified as intergenic transcripts, divergent transcripts adjacent to coding transcripts on the opposite strand, anti-sense transcripts of protein-coding genes, and enhancer RNAs (eRNAs) that are transcribed near enhancers (**Figure 1**) (32). In addition, alternative splicing generates multiple isoforms of the same lncRNA, which may affect its subcellular localization and functions. In general, lncRNAs show tissue-specific expression patterns and are expressed in low abundance compared to protein-coding genes, except for some highly expressed lncRNAs such as *MALAT1* (Metastasis-associated lung carcinoma transcript 1) (22, 33).

**Figure 2:**
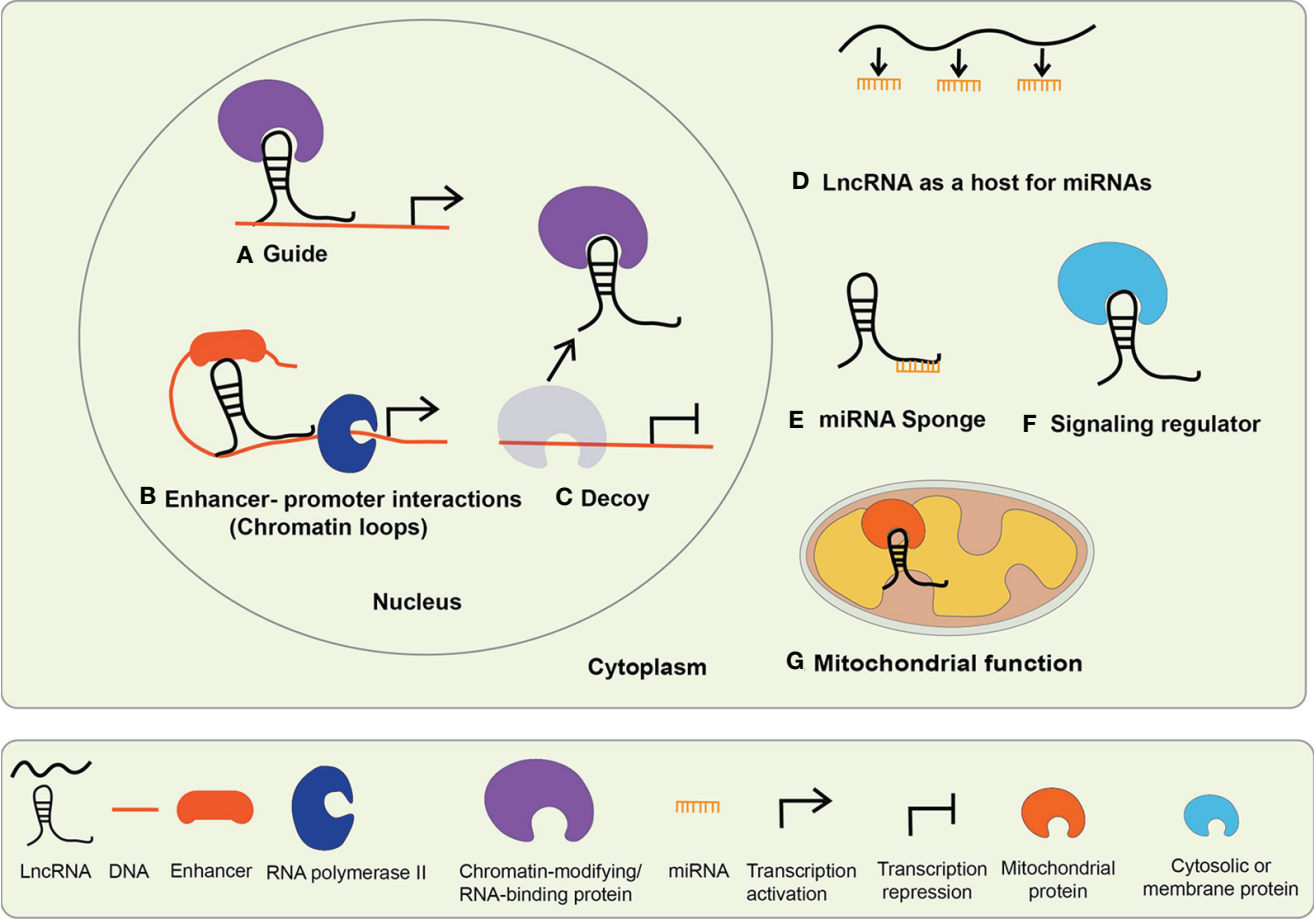
Mechanisms of lncRNA mediated gene regulation. The mechanisms of lncRNA actions are dependent upon their subcellular localization. LncRNAs localized in the nucleus mediate transcriptional regulation, whereas those located in the cytoplasm regulate *via* post-transcriptional mechanisms. Nuclear lncRNAs regulate transcription by **(A)** acting as guide lncRNAs that interact with chromatin-modifying and RNA-binding proteins to facilitate their recruitment on regulatory DNA sequences to activate or repress gene expression; **(B)** mediating enhancer promoter interactions-LncRNAs promote long-range enhancer-promoter interactions *via* chromatin looping; **(C)** acting as decoys that interact with the regulatory proteins to sequester them and prevent their normal functions. Cytosolic lncRNA mechanism of action includes: **(D)** serving as host genes for miRNAs, i.e. they harbor miRNAs within their exonic or intronic sequences; **(E)** acting as miRNA Sponges or competitive endogenous RNA (ceRNA)s, compete with mRNAs for shared complementary miRNA binding sites; **(F)** interacting with cytosolic or membrane proteins to regulate their functions and signaling processes; **(G)** serving as mitochondrial protein activators-lncRNAs interact with key mitochondrial proteins to modulate mitochondrial functions such as fatty acid oxidation.

The functions and mechanisms of actions of lncRNAs generally depend on their subcellular localization. Biochemical and epigenomic approaches have revealed that lncRNAs regulate genes *via* transcriptional and post-transcriptional mechanisms (32, 34–36). Nuclear lncRNAs can regulate epigenetic mechanisms involved in gene transcription and function of enhancers *via* interactions with chromatin, chromatin regulatory proteins, other RNA binding proteins, and TFs (**Figure 2**) (17, 32). On the other hand, cytoplasmic lncRNAs can alter mRNA stability or translation, regulate the expression and function of miRNAs and their targets by acting as sponges for miRNAs, and influence the actions of signaling proteins (**Figure 2**) (32, 35). It is now well established that lncRNAs are involved in many biological processes like cell proliferation, differentiation, inflammatory and immune responses (37). Emerging evidence shows that dysregulated expression and functions of lncRNAs can modulate pathologic gene expression associated with diabetes and its complications, including cardiovascular diseases (CVDs), DN, DCM, and DR (18, 21).

## Role of LncRNAs in Inflammatory Processes and Diabetic Cardiovascular Complications

Chronic inflammation is associated with both diabetes as well as most of the associated complications, including CVDs (2, 7). Monocytes and macrophages are innate immune cells that regulate inflammatory responses and host defense against invading microbial pathogens (38, 39). After acute exposure to environmental and metabolic cues like HG, FFAs, viruses, or endotoxin, these cells respond by inducing inflammatory cytokines *via* activation of nuclear factor kappa B (NF-κB) TF mediated pathways. This inflammatory phase is normally followed by an inflammation resolution phase (40, 41). However, recurrent exposure to these insults can weaken the inflammation resolution pathways leading to chronic inflammation associated with multiple disease conditions, including diabetes and its complications (41–43). Different stages of inflammation and inflammation resolution are tightly regulated by distinct gene expression patterns and phenotypic modulation of monocytes and macrophages *via* complex interactions between cis-regulatory elements on chromatin and transcription factors/regulators (44–46). Alterations in these transcription programs and dysregulated expression of inflammatory genes contribute to uncontrolled inflammation and diabetic vascular complications.

**Figure 3:**
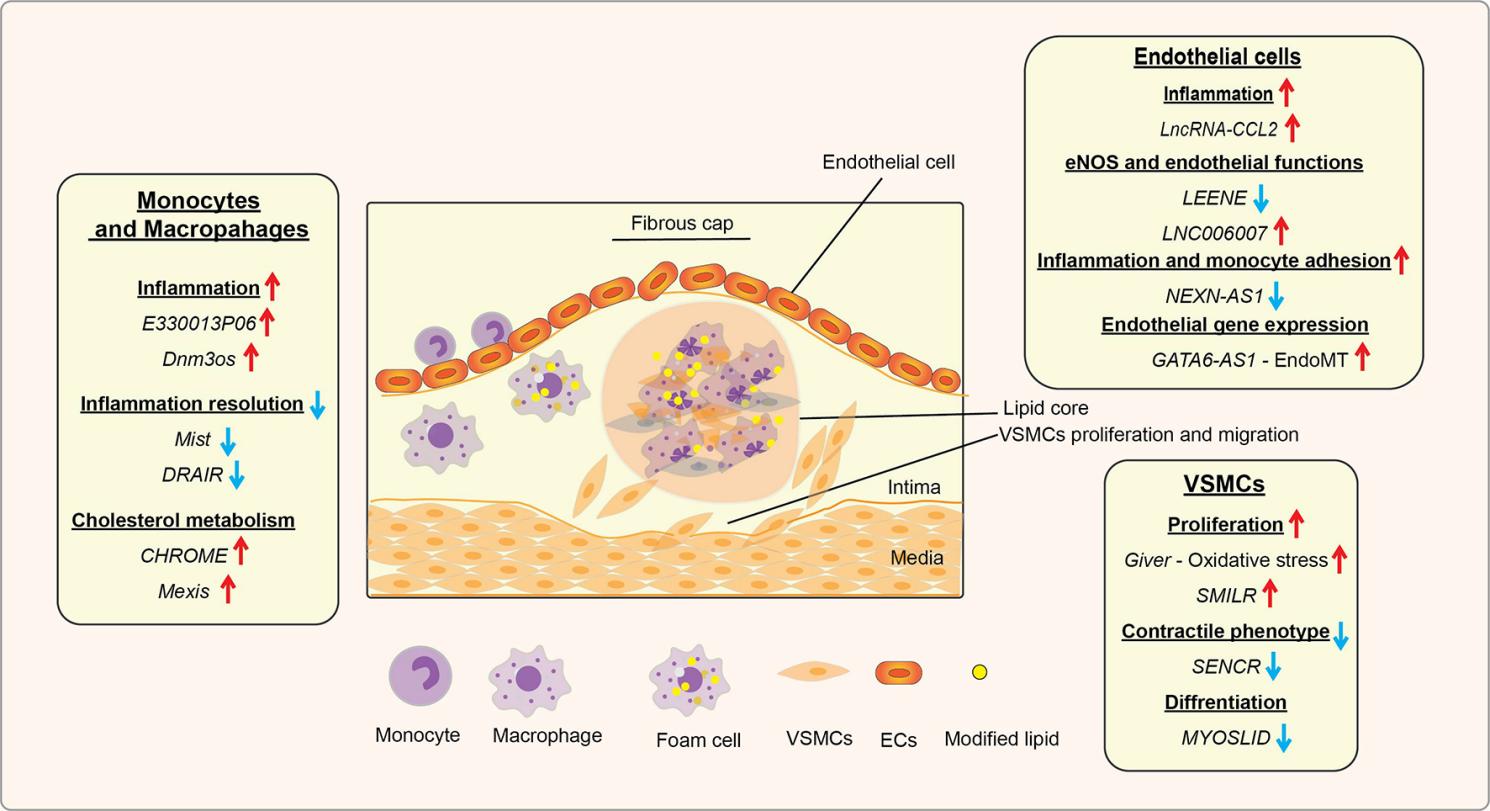
Role of lncRNAs in inflammatory processes and diabetic cardiovascular complications. A schematic showing the roles of candidate lncRNAs with functions in endothelial cells (ECs), monocytes and macrophages, and vascular smooth muscle cells (VSMCs) during the initiation and progression of inflammatory CVDs, such as atherosclerosis, that are enhanced in diabetes. The reported roles of lncRNAs in processes related to atherosclerosis and related CVDs are indicated under relevant cell types (monocytes/macrophages, ECs, and VSMC). Arrows indicate the direction of changes in the expression or function. CVDs, Cardiovascular diseases; EndoMT, Endothelial to mesenchymal transition.

In diabetes, increases in levels of deleterious lipids and FFAs in the circulation along with HG and growth factors like Angiotensin II (AngII) accelerate the risk for CVDs, including hypertension and atherosclerosis (47, 48). Hyperglycemia and these atherogenic factors promote inflammation in monocytes/macrophages, vascular smooth muscles, and endothelial cells, endothelial dysfunction, trans-endothelial migration of monocytes, foam cell formation, and VSMC migration and proliferation, key events in hypertension, restenosis, and atherosclerotic lesion formation (47). In advanced stages of atherosclerosis, macrophage apoptosis, defective efferocytosis, and dysregulated extracellular matrix (ECM) deposition in VSMCs play important roles in plaque stability (47). In diabetes, all these processes are significantly enhanced and result in accelerated atherosclerosis and unstable plaques, which are prone to rupture and thrombosis (48). Accumulating bodies of evidence show that a number lncRNAs are involved in the dysregulated functions of monocytes, macrophages, endothelial cells, and VSMCs associated with inflammation and the pathogenesis of atherosclerosis under diabetic or non-diabetic states (**Table 1** and **Figure 3**) (21, 86–88), suggesting their potential use as biomarkers and therapeutic targets. The following sub-sections highlight recent advances in the roles played by key candidate lncRNAs expressed in these inflammatory and vascular cells in processes related to diabetic CVDs.

**Table 1:** Long non-coding RNAs and their functions in inflammation and diabetes complications.

LncRNAs	Function	Cells/Tissue/Model	References
*Dnm3os*	Increases inflammation and phagocytosis	Macrophages	(27)
		monocytes	
*E330013P06*	Increases inflammation and foam cell formation	Macrophages	(49)
		monocytes	
*Lethe*	Decreases inflammation	Macrophages	(50, 51)
		Mouse embryonic fibroblasts	
*Mist*	Anti-inflammatory response	Macrophages and Human adipose tissue	(29)
*DRAIR*	Anti-inflammatory response	Monocytes and Macrophages	(52)
*CHROME*	Cholesterol metabolism	Plasma and atherosclerotic plaques, human hepatocytes, monocytes, and macrophages	(53)
*MeXis*	Regulates macrophage cholesterol efflux and atherogenesis	Macrophages	(54)
*LncRNA-CCL2*	Regulates expression of *CCL2*	Vascular ECs, human umbilical vein ECs (HUVECs)	(55)
*LINC00607*	Promotes endothelial dysfunction	Vascular endothelial cells HUVECs	(56)
*LEENE*	Regulates endothelial nitric oxide synthase and endothelial function	HUVECs	(57)
*STEEL*	Promotes angiogenesis	HUVECs	(58)
*NEXN-AS1*	Inhibits inflammation and monocyte adhesion *via* NEXN upregulation	HUVECs, monocytes, and VSMCs	(59)
*MANTIS*	Angiogenesis	HUVECs	(60)
*GATA6-AS*	Endothelial to mesenchymal transition phenotype	HUVECs	(61)
*Giver*	Increases inflammation, proliferation, and oxidative stress	VSMCs	(28)
*SMILR*	Controls cell cycle and VSMC proliferation	HSVSMCs	(62)
(RP11-94A24.1)		HCASMCs	
*MIAT*	Increase inflammation, apoptosis, and fibrosis	Cardiac tissue and cardiomyocytes	(63)
*CRNDE*	Decreases fibrosis	Heart tissue and cardio fibroblast	(64)
*TUG1*	Increases cardiac hypertrophy	cardiomyocytes	(65)
*H19*	Increases left ventricular hypertrophy	Cardiomyocytes	(66)
*LIPCAR*	Cardiac remodeling and diastolic function	Plasma of heart failure patients	(67, 68)
*Meg3*	ECM deposition and cardiac hypertrophy	Cardiac fibroblasts	(69, 70)
*Chaer*	Cardiac hypertrophy	Cardiomyocytes and fibroblasts	(71)
*Lnc-MGC*	Promotes ECM accumulation, hypertrophy	Mouse models of DN, mouse and human mesangial cells	(72)
	ER stress and DN phenotypes		
*TUG1*	Mitochondrial biogenesis	Mouse podocytes	(73, 74)
*LRNA9884*	Renal inflammation	Murine kidney proximal tubular epithelial cells	(75)
*PVT1*	Increases fibrotic genes	Mesangial cells	(76)
*Erbb4-IR*	Increases fibrotic genes	Mesangial cells	(77)
		Tubular epithelial cells	
*MALAT1*	Glomerular endothelial cell injury	Human renal glomerular endothelial cells	(78)
*MALAT1*	Inflammatory response	Retinal tissues	(79, 80)
	Oxidative stress	Retinal endothelial cells	
*MIAT*	Increases apoptosis and inflammation	Retinal endothelial cells	(81)
*H19*	Endothelial to mesenchymal transition phenotype	Retinal endothelial cells	(82)
*Anril*	Retinal tissue inflammation	Retinal tissue	(83)
*MEG3*	Retinal tissue inflammation	Retinal tissue and serum	(84, 85)

ECs, endothelial cells; ECM, extracellular matrix; ER, endoplasmic reticulum; Ldlr^−/−^, Mice homozygous for the Ldlr^tm1Her^ mutation; VSMCs, vascular smooth muscle cells; HSVSMCs, human saphenous vein smooth muscle cells; HCASMCs, human coronary artery smooth muscle cells.

### LncRNA Functions in Monocytes and Macrophages

Several studies have shown that lncRNA mediated mechanisms regulate several key monocyte/macrophage functions associated with differentiation, inflammation, and innate immunity (89, 90). Since increased infiltration of inflammatory monocytes/macrophages into target organs and enhanced inflammation are key features of most diabetic vascular complications, lncRNAs that affect the expression of inflammatory genes in these cells are likely to be involved in diabetes, obesity, and diabetic CVDs. RNA-sequencing identified several dysregulated lncRNAs in bone marrow-derived macrophages (BMDM) from type 2 diabetic (T2D) db/db mice relative to control db/+ mice, and the function of one of these lncRNAs *E330013P06* (*E33*) was further characterized (49). Expression of the lncRNA *E33* was upregulated in macrophages from db/db mice, and high-fat diet (HFD) plus streptozotocin (STZ) induced T2D mice but not in STZ-induced type 1 diabetic (T1D) mice, suggesting a T2D disease-specific regulation of lncRNA *E33* (49). Furthermore, diabetogenic factors, like HG and palmitic acid (PA), upregulated lncRNA *E33* expression *in vitro* in mouse macrophages. Over-expression of the lncRNA *E33* increased the expression of inflammatory genes *Il6*, *Tnf*, *Ptgs2*, and *Ccl2* and promoted foam-cell formation (proatherogenic phenotype) in mouse macrophages. In contrast, siRNA-mediated knockdown of lncRNA *E33* inhibited HG and PA-induced upregulation of these inflammatory genes in primary mouse BMDM. Interestingly, the human ortholog of lncRNA *E33* was also upregulated in CD14^+^ monocytes from T2D patients. These results suggest that lncRNA *E33* is an important regulator of inflammation and foam cell formation in diabetes and diabetic vascular complications, and its functions are conserved in humans and mice.

Another lncRNA, Dynamin3 opposite strand (*Dnm3os*), was also induced in macrophages from T2D mice, HFD-induced insulin-resistant mice, T1D mice, and diabetic *Apoe^−/−^* mice (a mouse model of accelerated atherosclerosis), indicating that *Dnm3os* plays a key role in diabetes and accelerated atherosclerosis. *Dnm3os* was upregulated by PA *via* activation of NF-κB, supporting its pro-inflammatory properties (27). Stable overexpression of *Dnm3os* increased basal and PA-induced *Il6* and *Tnf* genes in mouse RAW macrophages. Furthermore, *Dnm3os* overexpression upregulated gene networks associated with inflammation, immune response, chemotaxis, phagocytosis, and wound healing, key processes associated with CVDs and diabetic vascular complications. Conversely, *Dnm3os* knockdown with siRNAs in macrophages from diabetic db/db mice ameliorated the enhanced expression of inflammatory genes (*Il6*, *Tnf*, *Itgax*, and *Nfkbiz*) and phagocytosis of *E.coli* bioparticles, clearly supporting its role in diabetes-induced inflammation. Further, mechanistic studies showed that *Dnm3os* overexpression increased global levels of chromatin marks such as histone H3K9ac and H3K27ac associated with active transcription, suggesting it may act *via* epigenetic mechanisms.

Additionally, RNA-pulldown assays and RNA-fluorescence *in-situ* hybridization (RNA-FISH) revealed that *Dnm3os* interacts with the multifunctional nucleolar protein nucleolin, which is known to have anti-inflammatory and protective roles in macrophages. Nucleolin protein levels were also reduced in macrophages treated with HG and PA and in macrophages of T2D db/db mice. Further studies with nucleolin knockdown and ChIP assays suggested that, under normal conditions, the interaction with nucleolin restrains the actions of *Dnm3os* and maintains compact chromatin near inflammatory gene promoters. However, under diabetic conditions, the altered levels of *Dnm3os* and nucleolin disrupt this homeostasis, and augments the interaction of *Dnm3os* with histone acetyltransferases, leading to H3K9ac enrichment, chromatin relaxation, and increased inflammatory gene expression. Further highlighting its relevance, human *DNM3OS* levels were increased in CD14^+^ monocytes from T2D patients and *in vitro* in PA-treated macrophages derived from CD14^+^ monocytes from non-diabetic humans. Moreover, GapmeR-mediated knockdown of human *DNM3OS* in human THP1 monocytes inhibited inflammatory genes (*IL6*, *TNF*, and *ITGAX*) and phagocytosis similar to actions of the mouse ortholog, suggesting *DNM3OS* upregulation in humans likely contributes to enhanced inflammation in diabetes. However, *Dnm3os* levels have not been examined in endothelial cells (ECs) and VSMCs in diabetes.

Resolution of the inflammatory response is required for normal homeostasis, tissue repair, and wound healing (91, 92). Apart from increases in pro-inflammatory lncRNAs, reductions of “protective” lncRNAs that restrain, or mediate inflammation resolution could also contribute to enhanced inflammation and impaired wound healing in diabetes. LncRNA *Lethe* (named after the mythological river of forgetfulness for its role in negative feedback) was induced by pro-inflammatory cytokines (TNF-α and IL-1β) and anti-inflammatory dexamethasone in mouse embryonic fibroblasts (50). *Lethe*, a chromatin-associated lncRNA, suppressed inflammatory gene expression by inhibiting the DNA binding activity of RelA (p65), the active subunit of NF-κB (50). Interestingly, *Lethe* expression was downregulated while the oxidative stress gene NADPH oxidase 2 (*Nox2*) was upregulated by HG in mouse macrophages. Overexpression of *Lethe* attenuated HG-induced *Nox2* and reactive oxygen species (ROS) production as well as NF-κB activation in macrophages. Furthermore, *Lethe* expression was downregulated *in vivo* in peritoneal macrophages, BMDM and in wounds from T2D db/db mice, suggesting a potential protective role for *Lethe* against impaired wound healing in diabetes (51).

Similarly, an anti-inflammatory function was demonstrated for a novel lncRNA Macrophage Inflammation-Suppressing Transcript (*Mist)* in macrophages during obesity. *Mist* was one of the lncRNAs differentially expressed in peritoneal macrophages (PMs) from HFD-induced obese insulin-resistant mice relative to control-diet fed mice (29). LncRNA *Mist* and its adjacent coding gene *Fabp5* were both downregulated in PMs and adipose tissue macrophages from HFD-fed obese mice. *Mist* was found to inhibit the expression of inflammatory genes *Tnf*, *Il1b Nos2*, and *Il6*, and foam cell formation in mouse macrophages. Mechanistic studies, including RNA pull-down coupled with mass spectrometry, showed that *Mist* interacts with Poly (ADP)-ribose polymerase-1 (PARP1) protein. Disruption of this interaction by Gapmer mediated knockdown of *Mist* led to increased enrichment of PARP1 and chromatin PARylation, a permissive epigenetic modification, at the *Il6* and *Tnf* promoters and their upregulation. Expression of the human ortholog *MIST* was also downregulated by pro-inflammatory stimuli, and its expression in human adipose stromal vascular fractions (enriched in macrophages) inversely correlated with obesity, insulin resistance, and metabolic dysfunction (29). Together these results supported the notion that *MIST* has anti-inflammatory properties and its downregulation in obesity contributes to augmented inflammation.

Furthermore, lncRNAs can also modulate monocyte activation associated with chronic inflammation in human T2D. Recently, RNA-seq analysis of CD14^+^ monocytes from T2D patients *versus* healthy controls revealed dysregulated expression of several lncRNAs along with reduced levels of protective genes in T2D, which could be associated with enhanced monocytosis and inflammatory phenotype of monocytes. Interestingly, a novel lncRNA *DRAIR* (Diabetes Regulated anti-inflammatory RNA) was found to be downregulated in T2D monocytes. The lncRNA *DRAIR* is divergently expressed adjacent to Cytoplasmic polyadenylation element-binding protein 2 gene (*CPEB2)*, which belongs to CPEB family of proteins with anti-inflammatory functions (93), suggesting a potential role for *DRAIR* in inflammation. Furthermore, HG and PA downregulated *DRAIR* in CD14^+^monocytes from healthy normal controls. On the other hand, *DRAIR* was upregulated by anti-inflammatory cytokines IL-4 and IL-13 *via* KLF4, a key transcription factor that promotes anti-inflammatory phenotype in macrophages. Gain- and loss-of-function experiments showed that *DRAIR* increases anti-inflammatory genes like *IL1RN* and macrophage differentiation markers like CD36, whereas *DRAIR* inhibits pro-inflammatory *TNF* and *IL1B*, and EC-monocyte binding and phagocytosis, supporting anti-inflammatory functions for *DRAIR*. Mouse orthologous *Drair* was also downregulated in T2D db/db mice and its knockdown with locked nucleic acid (LNA) modified GapmeRs in macrophages *in vitro* and *in vivo* in non-diabetic mice enhanced *Tnf*, *Il1b*, and *Il6* genes. Further studies with Chromatin Isolation by RNA Purification assays (ChIRP-seq) and ChIRP-Mass spectrometry revealed that *DRAIR* interacts with chromatin and chromatin-modifying proteins, including G9a, which mediates H3K9me2. Moreover, G9a was upregulated in monocytes from T2D subjects and in human monocytes treated with HG+PA, and its knockdown with siRNAs increased anti-inflammatory genes. Furthermore, *DRAIR* overexpression increased anti-inflammatory genes *via* inhibition of G9a recruitment and enrichment of repressive H3K9me2 at their promoters. These studies suggest that inhibition of *DRAIR* mediated epigenetic mechanisms *via* its downregulation and increased G9a could contribute to monocyte dysfunction and inflammation in diabetes (52).

Hyperlipidemia, characterized by high cholesterol, LDL, and triglyceride levels, is a strong driver of atherosclerosis in animal models and humans and is associated with high cholesterol diet and diabetes. Macrophages from hyperlipidemic *Ldlr* ^−/−^ mice and *Apoe* ^−/−^ mice fail to effectively clear LDL from the circulation leading to the development of spontaneous atherosclerotic lesions. Nuclear receptors such as the liver X receptor (LXR) family play key roles in the regulation of genes involved in cholesterol homeostasis (94). LncRNAs regulated by LXR such as *CHROME* (Cholesterol Homeostasis Regulator of MiRNA Expression) and *MeXis* (Macrophage-expressed LXR-induced sequence) were shown to regulate cholesterol metabolism genes in macrophages (**Table 1** and **Figure 3**) (53, 54). *MeXis* was identified as an amplifier of LXR-dependent transcription of *Abca1*, which plays a critical role in regulating cholesterol efflux. Additionally, *CHROME* was shown to promote cholesterol efflux and HDL biogenesis by restraining key functionally related miRNAs that downregulate important genes (including *Abca1*) related to cholesterol homeostasis and atherosclerosis (53, 54). However, further studies are needed to extend these findings to diabetic CVDs.

Together these reports show that lncRNAs in monocytes/macrophages can regulate inflammatory processes and lipid metabolism *via* interactions with chromatin factors and signaling proteins to modulate the epigenetic states at the promoters of key genes associated with CVDs in diabetes. Further characterization of such lncRNAs in humans could lead to their utility as novel biomarkers for early detection and/or therapeutic targets to reduce inflammation, promote cholesterol homeostasis, or inhibit the accelerated progression of CVDs, like atherosclerosis and hypertension in diabetes.

### LncRNA Functions in Endothelial Cells

The endothelium makes up the innermost layer of the vasculature, and disruption of endothelial homeostasis leads to endothelial dysfunction in diabetes and several diabetic vascular complications, including CVDs (2, 47). In addition, many lncRNAs implicated in EC homeostasis are regulated by local mechanical, hypoxic, and inflammatory conditions associated with CVD (86).

Atherosclerotic lesions are more localized in the aortic arch where disturbed blood flow patterns promote atherogenic genes and EC apoptosis. By examining the regulatory role of blood flow-dependent lncRNAs expressed in EC can provide new insights into EC functions, and several lncRNAs are regulated by changes in mechanical (hemodynamic) forces (57, 58). Nitric oxide (NO) produced by endothelial nitric oxide synthase (eNOS) has vital vasodilatory functions and is critical for EC homeostasis. Pathological flow patterns and inflammatory conditions involved in CVDs downregulate eNOS (*NOS3*) *via* epigenetic mechanisms (95). ECs subjected to physiological flow increased not only *eNOS*, but also a lncRNA *LEENE* (lncRNA that enhances *eNOS* expression) an eRNA expressed from a nearby enhancer, in KLF2 and KLF4 dependent manner (57). *LEENE* upregulated *eNOS* expression *via* epigenetic mechanisms such as promoting enhancer-promoter interactions and recruitment of RNA pol II at the *eNOS* promoter (57). Moreover, *LEENE* knockdown increased monocyte-EC adhesion further supporting its anti-inflammatory functions. Similar functions and regulation were also exhibited by a mouse ortholog *BY707159.1* suggesting conserved functions across species. Another EC enriched nuclear lncRNA, Spliced-transcript endothelial-enriched lncRNA (*STEEL*), was shown to regulate several genes involved in EC functions, including angiogenesis (58). *STEEL* also induced the expression of KLF2 and its target *eNOS via* increasing *eNOS* promoter recruitment of PARP1, which is implicated in EC dysfunction (58). The authors suggested that *STEEL* plays an important role in transcriptional regulation of EC identity. Furthermore, a search for epigenetically regulated lncRNAs by shear stress in ECs led to the discovery of lncRNA *MANTIS*, which was regulated by a histone demethylase JARID1B (60). *MANTIS*, a nuclear lncRNA, was shown to regulate the expression of genes associated with angiogenesis *via* interaction with BRG1, the catalytic subunit of the SWI/SNF complex, promoting chromatin remodeling and recruitment of RNA pol II. *MANTIS* expression was downregulated in idiopathic pulmonary hypertension in humans and rats and increased during atherosclerosis regression in primates, suggesting it might have protective functions in ECs (60).

In addition to hemodynamic forces, hypoxia was also shown to regulate lncRNAs such as lncRNA *GATA6-AS* in ECs (61). *GATA6-AS* could promote endothelial-mesenchymal transition and regulate hypoxia as well as angiogenesis-related genes, including *PTGS2* and *POSTN* implicated in the development of CVDs (61). This study suggested that *GATA6-AS* regulates angiogenesis-related genes and endothelial function *via* epigenetic mechanisms, at least in part, by binding to a fraction of nuclear Lysyl oxidase-like 2 (LOXL2) *in vitro* and *in vivo* experiments. LOXL2 was identified as a transcription co-repressor that reduces the permissive histone modification H3K4me3 *via* deamination of the trimethylated lysine 4 (96). Interaction with *GATA6-AS* was shown to inhibit LOXL2 function and increase H3K4me3 levels at angiogenesis-related target gene promoters.

*lncRNA-CCL2* and *LINC00607* are involved in regulating inflammation in human umbilical vein ECs (HUVECs) (55, 56). *LncRNA-CCL2*, a divergent transcript expressed adjacent to *CCL2*, was induced by the pro-inflammatory cytokine IL-1β in primary ECs, immortalized HUVECs and dermal microvascular ECs. In addition, *LncRNA-CCL2* upregulated *CCL2*, which codes for chemokine CCL2 involved in inflammatory cell recruitment *via* post-transcriptional mechanisms involving interactions with RNA binding proteins (55). Moreover, increased *lncRNA-CCL2* levels correlated with increased *CCL2* levels in primary HUVECs treated with IL-1β and unstable symptomatic human atherosclerotic plaques.

Emerging studies show that chromatin-associated lncRNAs can also have functional roles in ECs. An integrative Omics study characterized HG plus TNF-α induced changes in ECs using a combination of single-cell RNA-seq, Hi-C (to identify DNA-DNA interactions), and *in situ* mapping of RNA–genome interactome (iMARGI) (to identify RNA-chromatin interactions genome-wide). HG + TNF-α treatment induced inter-chromosomal RNA-chromatin interactions that were particularly clustered around super enhancers from many chromosomes (56). One of the key chromatin-associated lncRNAs induced by HG and TNF-α, *LINC00607*, regulated *SERPINE1*, a known pro-inflammatory and pro-fibrotic gene. Furthermore, bioinformatics analysis of the co-expression gene networks in arterial ECs obtained from diabetic *versus* control human donors could closely substantiate HG + TNF-α induced RNA-chromatin interactions *in vitro*. This report highlights the putative role of chromatin-associated RNAs in regulating genes associated with EC dysfunction under diabetic conditions.

Another nuclear lncRNA *NEXN-AS1* and nearby gene *NEXN* (Nexilin F-actin-binding protein) were downregulated in atherosclerotic plaques from humans (59). *NEXN-AS1* was shown to upregulate *NEXN*, an atheroprotective gene, *via* interacting with chromatin upstream of *NEXN* promoter and inhibiting the transcription repressor BAZ1A (also known as ATP-utilizing chromatin assembly and remodeling factor 1 [ACF1]) to promote open chromatin formation. *NEXN* and *NEXN-AS1* knockdown enhanced pro-inflammatory genes such as *CCL2*, *TNF*, and *IL6* in ECs. Furthermore, heterozygous *NEXN* ^+/−^ Apoe^−/−^ mice on Western diet showed significantly more atherosclerosis relative to *NEXN* ^+/+^ Apoe^−/−^ mice, suggesting that *NEXN-AS1* mediates atheroprotective effects *via NEXN* regulation (59).

Overall, these reports clearly show that lncRNAs are important epigenetic regulators of endothelial function and could therefore be valuable therapeutic targets for CVDs. It is yet to be determined if dysregulation of many of these lncRNAs is associated with accelerated vascular inflammation, EC differentiation and dysfunction in diabetes/metabolic disease.

### LncRNA Functions in VSMCs

Evidence shows that lncRNAs can also modulate various VSMC functions, including phenotypic switching, proliferation, oxidative stress, and inflammation-key processes associated with accelerated CVDs and other vascular complications of diabetes. Several known and novel lncRNAs that are regulated by growth factors, inflammatory cytokines and in CVDs have been identified using sequencing approaches in rat and human VSMCs (28, 88, 97). VSMC lncRNAs were identified for the first time by integration of datasets from RNA-seq and ChIP-seq (H3K4me3/H3K36me3) from rat VSMCs treated with vehicle or Angiotensin II (AngII), a potent VSMC growth factor that promotes hypertension and atherosclerosis (30). AngII-induced differential expression of 491 lncRNAs, including *Lnc-Ang362*, which is co-transcribed with miRNAs miR-221 and miR-222 that are implicated in VSMC proliferation (98). Accordingly, *Lnc-Ang362* knockdown with siRNAs downregulated miR-222 and miR-221 and inhibited VSMC proliferation, demonstrating that non-coding RNAs can play functional roles in AngII-induced VSMC dysfunction related to CVDs.

A novel lncRNA *Giver* (growth factor- and proinflammatory cytokine-induced vascular cell-expressed RNA) located near the nuclear receptor *Nr4a3* was also markedly induced by AngII, growth factors, HG, and inflammatory cytokines (28). Further studies revealed that *Giver* is a nuclear lncRNA, regulated by the nuclear receptor Nr4A3 and promoted oxidative stress, inflammatory gene expression, and proliferation in VSMC. *Giver*-induced gene expression was mediated, at least in part, by its interaction with several nuclear proteins, such as NONO (Non-POU Domain Containing Octamer Binding) protein and regulation of epigenetic histone PTMs at target genes. Interestingly, human *GIVER* was upregulated in arteries from hypertensive patients but significantly attenuated in hypertensive patients taking ACE (angiotensin-converting enzyme) inhibitors and angiotensin receptor blockers, supporting a role for *GIVER* in AngII signaling and hypertension.

Another VSMC-specific lncRNA, Smooth Muscle Enriched Long Non-coding RNA (*SMILR*), was identified using RNA-seq of human VSMCs treated with platelet derived growth factor (PDGF) and inflammatory cytokine (IL-1β) (62). Knockdown of *SMILR* decreased VSMC proliferation and conversely, its overexpression increased VSMC proliferation. *SMILR*, a cytoplasmic enriched lncRNA, was found to regulate the late phase of the mitotic cycle *via* interaction with mRNA of a key mitotic centromere protein and RNA binding protein Staufen1. Moreover, *SMILR* was upregulated in unstable human atherosclerotic plaques and positively correlated with elevated levels of inflammatory C-reactive protein, suggesting that targeting *SMILR* may confer protection from atherosclerosis. VSMC de-differentiation and phenotypic switching play key roles in VSMC dysfunction and CVDs like hypertension and atherosclerosis. Smooth muscle and endothelial cell-enriched migration/differentiation-associated long non-coding RNA (*SENCR*) was one of the first lncRNAs shown to be expressed in both human ECs and VSMCs. *SENCR* could stabilize VSMC contractile phenotype and inhibit migration, suggesting it has protective functions in the vasculature (97). More recently, another lncRNA MYOcardin-induced Smooth muscle LncRNA, Inducer of Differentiation (*MYOSLID*) was reported to be the first VSMC-selective lncRNA that acts as a novel amplifier of the VSMC differentiation program *via* distinct mechanisms. Furthermore, *MYOSLID* expression was reduced in CVD, supporting a vasculo-protective role (99).

Together these studies show lncRNAs act *via* novel (including epigenetic) mechanisms in VSMCs and have important functional roles related CVDs that could be exploited for therapy (**Table 1** and **Figure 3**).

## Role of LncRNAs in Diabetic Cardiomyopathy (DCM)

**Figure 4:**
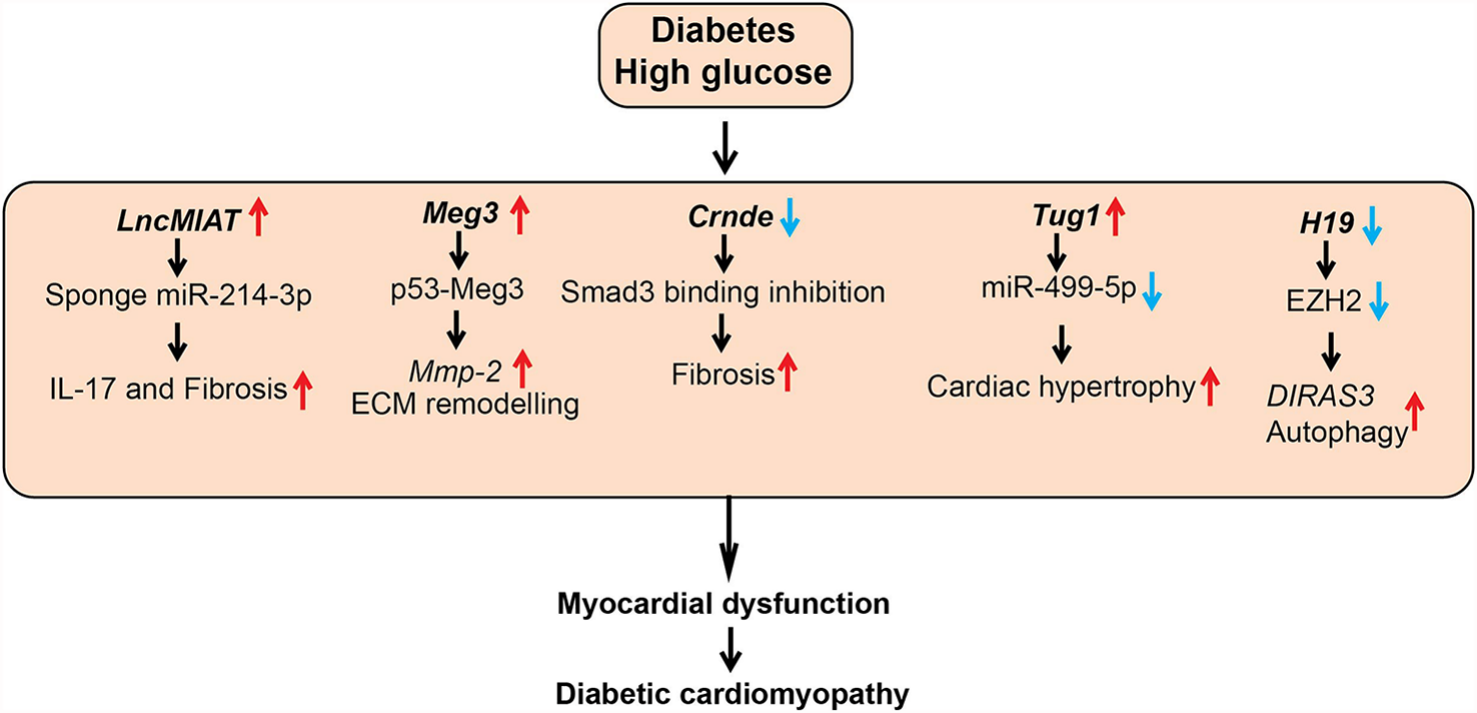
Role of lncRNAs in diabetic cardiomyopathy (DCM). A schematic showing dysregulated function of lncRNAs in the pathogenesis of DCM, such as cardiac hypertrophy, fibrosis, and inflammation. Arrows indicate the direction of changes in the expression or function. *Mmp-2*, Matrix Metalloproteinase-2; ECM, Extracellular matrix; Smad3, SMAD family member 3; EZH2, Enhancer of Zeste 2 Polycomb Repressive Complex 2 Subunit; DIRAS3, DIRAS Family GTPase 3.

Diabetic cardiomyopathy (DCM) is also a common and severe complication of diabetes characterized by myocardium dysfunction, cardiac fibrosis, and microvascular abnormalities. DCM occurs even in the absence of traditional risk factors like hypertension and coronary artery disease (100). Factors associated with diabetes, including HG, AGEs and lipids, increase myocardial fibrosis, cardiomyocyte hypertrophy and stiffness, and inflammation, which contribute to DCM (100). Accumulating evidence suggests that lncRNAs play a key role in DCM (**Table 1** and **Figure 4**) (101).

A number of studies investigated the role of the lncRNA Myocardial Infarction Associated Transcript (*MIAT*) in DCM. *MIAT* was identified as a susceptible locus for myocardial infarction on human chromosome 22q12 (102). *MIAT* levels were upregulated in cardiac tissues and serum from patients with DCM and in animal models of DCM along with several inflammatory cytokines, including IL-17, IL-1β, IL-6, and TNF-α. HG upregulated *MIAT* and these inflammatory cytokines and promoted apoptosis in cardiomyocytes. HG-effects were inhibited by siRNA-mediated knockdown of *MIAT*, but they were augmented by *MIAT* overexpression. Mechanistic studies showed that *MIAT* acts as a competing endogenous RNA to inhibit functions of miRNAs, such as miR-214-3p and miR-22-3p, that target inflammatory and apoptosis genes. Furthermore, lentiviral-based knockdown of *MIAT* in diabetic animals improved cardiac ejection fraction, decreased inflammatory cytokines (IL-17, IL-6, IL-1β, and TNF- α) and fibrotic markers (collagen I and III) in heart tissue (left ventricle), inhibited apoptosis, and ameliorated DCM (63, 103).

Expression of another lncRNA, colorectal neoplasia differentially expressed (*CRNDE*), was also upregulated and negatively correlated with the myocardial fibrosis markers, including *COL1A1* in human heart tissue. *Crnde* expression was upregulated in cardiac fibroblasts treated with Transforming growth factor-beta1 (TGF-β1) and angiotensin II, and in heart tissues from a mice model of myocardial infarction. Adeno-Associated Virus (AAV)-mediated overexpression of *Crnde in vivo* in a mouse model of DCM decreased the deposition of fibrosis marker collagen in the hearts of the mice with DCM compared to control. Furthermore, *Crnde* overexpression in cardiac fibroblasts *in vitro* reduced TGF-β1 induced expression of myofibroblast markers (*Col1a1*, *Col3a1*, and *Acta2*). *Crnde* was found to inhibit the binding of SMAD family member 3 (Smad3) to Smad binding element and downregulate the expression of key myofibroblast marker genes such as *Acta2*. However, further studies are needed to determine if human *CRNDE* has function and mechanism of action similar to the murine ortholog (64).

Additionally, LncRNA *TUG1* (taurine upregulated gene1) was upregulated in cardiomyocytes of diabetic mice. Lentiviral-mediated *TUG1* knockdown (si-TUG1) improved diastolic dysfunction in a mice model of DCM but had no effect on hyperglycemia and dyslipidemia. *TUG1* inhibition also decreased the expression of key hypertrophic markers as well as fibrotic area in db/db mice. Furthermore, this study reported that *TUG1* mediates cardiac hypertrophy in DCM *via* inhibition of miR-499-5p (65). Dysregulated autophagy also promotes cardiac dysfunction in DCM, and the role of lncRNAs such as *H19* in autophagy has been reported. *H19* was downregulated in the myocardium of a rat model of DCM and lentiviral-mediated overexpression of *H19* decreased autophagy and improved left ventricular dysfunction. Furthermore, overexpression of *H19* decreased levels of diabetes-induced autophagy markers, such as LC3-II, BECN1, and ATG7, in cardiomyocytes. Mechanistic investigations demonstrated that *H19* represses autophagy inducer *DIRAS3 via* recruiting the repressive histone H3K27me3 methyltransferase EZH2 and increasing promoter H3K27me3. Thus, *H19* downregulation in diabetes can de-repress epigenetic mechanisms to upregulate *DIRAS3* and activate autophagy. Results from this study suggest that overexpression of *H19* could be an approach to alleviate progression of DCM (66).

LncRNA *Meg3* was reported to be enriched in cardiac fibroblasts (CFs) and dysregulated during pressure overload–induced cardiac remodeling. *Meg3* knockdown with GapmeRs led to dysregulation of several matrix metalloprotease (MMP)s in CFs. *Meg3*, a chromatin-associated lncRNA interacted with p53 and this interaction is required for the transcription of *Mmp2* promoter in CFs. Accordingly, *Meg3* knockdown reduced basal and TGF-β1-induced expression of *Mmp2* in CFs. Furthermore, GapmeR mediated knockdown of *Meg3 in vivo* decreased ECM deposition, cardiomyocyte hypertrophy and improved impaired diastolic function in a mice model of transverse aortic constriction-induced cardiac hypertrophy/heart failure (69). This study did not investigate the role of lncRNA *Meg3* in DCM. However, another study reported that HG upregulates *MEG3* expression in human cardiomyocytes, and *MEG3* knockdown attenuated HG-induced apoptosis. Their mechanistic studies revealed that *MEG3* binds to miR-145 and upregulates the miR-145 target *PDCD4*, which promotes apoptosis in cardiomyocytes. Overall these studies suggest that targeting *MEG3* may ameliorate DCM (70).

Another lncRNA *Chaer* (cardiac-hypertrophy-associated epigenetic regulator) was also found to be essential for the development of cardiac hypertrophy. *Chaer* induced cardiac hypertrophy by interacting with Ezh2 (component of the repressive PRC2 complex) *via* a 66-mer motif. This interaction inhibits PRC2 function and reduces deposition of repressive epigenetic mark H3K27me3 at the promoter regions of genes mediating hypertrophy. Inhibition of *Chaer* before the onset of pressure overload attenuated cardiac hypertrophy and dysfunction in mice. However, the role of lncRNA *Chaer* in DCM was not investigated (71).

Interestingly, circulating levels of the mitochondrial lncRNA *LIPCAR* (long intergenic non-coding RNA predicting cardiac remodeling) expression were downregulated early in patients with myocardial infarction but increased in later stages. *LIPCAR* expression was associated with chronic heart failure and its expression inversely correlated with diastolic function in T2D patients (67, 68). Furthermore, changes in circulating lncRNAs *MIAT* and *SENCR* were also associated with cardiac remodeling (i.e. changes in the size, shape, structure, and function of the heart) in patients with T2D (68). Overall, these reports support a role for lncRNAs in the regulation of fibrosis, inflammation, and hypertrophy in cardiac cells related to cardiac function. Further studies are needed to clarify utility of these lncRNAs as biomarkers and therapeutic targets in DCM.

## The Role of LncRNAs in Diabetic Nephropathy

**Figure 5:**
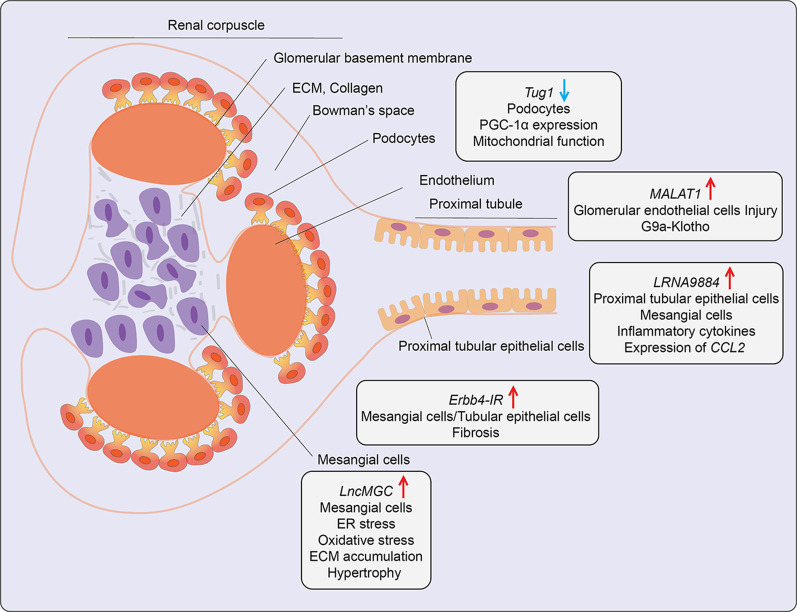
Role of LncRNAs in diabetic nephropathy (DN). A schematic showing the roles of candidate lncRNAs with functions in the indicated kidney cell types during development of diabetic nephropathy. Arrows indicate the direction of changes in the expression or function. ER, Endoplasmic Reticulum; CCL2, C-C Motif Chemokine Ligand 2; ECM, Extracellular matrix; G9a, Histone H3-lysine 9-dimethyl-transferase; PGC-1α, Peroxisome proliferator-activated receptor gamma coactivator 1-alpha.

Diabetic nephropathy (DN), a leading cause of renal failure, is characterized by micro- and macro-albuminuria and fibrosis in most renal cells and progressive renal dysfunction. Diabetes-induced HG and TGF-β1 regulate lncRNAs involved in DN. Often HG mediates its effect *via* TGF-β1, a potent profibrotic factor augmented in the diabetic kidney, and a major player in DN pathology. TGF-β1 induced activation of endoplasmic reticulum (ER) stress, PI3K/AKT signaling, and Smad TFs play key roles in expression of genes associated with DN (18). In recent years, multiple HG- and/or TGF-β1-induced lncRNAs have been shown to regulate genes associated with renal function, fibrosis, apoptosis, autophagy, inflammation, and ER stress in key affected renal cells like mesangial cells, tubular epithelial cells, endothelial cells, and podocytes, and therefore implicated in DN (18) (**Table 1** and **Figure 5**).

A lncRNA known as lncRNA megacluster (*lnc-MGC*) was found to play a key role in the pathogenesis of early DN (72). *Lnc-MGC* is a host gene for miR-379 cluster, *i. e.*, harbors miRNAs in its exonic and intronic sequences and these non-coding RNAs are present within one of the largest miRNA clusters in the genome. The expression of *lnc-MGC* and key component mature cluster miRNAs located within *lnc-MGC* were upregulated in the renal glomeruli of T1D and T2D mice (72). Both HG and TGF-β1 increased the expression of *lnc-MGC* and several cluster miRNAs located within it, such as miR-379, miR-494, miR-495, and miR-377 in mouse renal mesangial cells *via* the ER stress- related TF CHOP in collaboration with Smad2/3 TFs. The cluster miRNAs derived from *lnc-MGC*, including miR379 were observed to regulate target genes involved in various functions related to DN, such as protein synthesis, ER stress, RNA binding, and protein translation. Notably, targeting *lnc-MGC* using LNA-modified GapmeR antisense oligos *in vivo* in diabetic mice downregulated *lnc-MGC* and key cluster miRNAs, including miR-379, but upregulated miR-379 targets, such as ER Degradation Enhancer, Mannosidase Alpha-Like 3 (*Edem3*), a regulator of ER stress. In parallel, this GapmeR also ameliorated diabetes-induced expression of profibrotic genes *Tgfβ1*, *Col1a2*, *Col4a1*, and *Ctgf *in renal glomeruli and reduced key features of early DN in mice (72). Moreover, miR-379 knockout mice created with CRISPR-Cas9 editing were also protected from DN as shown by decreases in renal fibrosis, hypertrophy, albuminuria, and podocyte dysfunction (104). Since a GapmeR targeting human ortholog of *lnc-MGC* was effective in reducing fibrosis in human renal cells, these data highlight the translational potential of targeting pathological renal ncRNAs like *lnc-MGC* or miR-379 for the treatment of DN. In other studies, GWAS identified the association of lncRNA *PVT1*(plasmacytoma variant translocation1) locus with end-stage renal disease attributed to T1D and T2D (105, 106). Experimental studies demonstrated that lncRNA *PVT1* plays a key role in HG-induced fibrotic genes in mesangial cells, possibly *via* acting as host gene for miRNAs such as miR-1207-5p (76), further supporting the utility of targeting miRNAs hosted within lncRNAs for DN treatment.

Podocyte apoptosis and effacement are key pathological events associated with glomerular dysfunction and proteinuria in DN. The role of lncRNAs in podocyte dysfunction has been evaluated. One report demonstrated that lncRNA *Tug1* expression was downregulated in glomerular podocytes from kidneys of T2D db/db mice relative to control mice and in glomeruli from human subjects with DN (73). Further studies demonstrated that HG suppresses *Tug1* expression *via* recruitment of TF Carbohydrate response element-binding protein (ChREBP) and multiple co-repressors to the *Tug1* promoter (74). *Tug1* was mostly localized in the nucleus and upregulated peroxisome proliferator-activated receptor gamma coactivator 1α (PGC-1α), a master transcriptional regulator of mitochondrial biogenesis. *Tug1* was shown to regulate *Ppargc1a* expression through interaction with chromatin at *Tug1* binding sites in the upstream region (73). *Tug1* overexpression improved mitochondrial function in podocytes of diabetic mice. Thus, *Tug1* downregulation in diabetes appears to inhibit the expression of PGC-1α and its target genes involved in mitochondrial biogenesis, resulting in podocyte apoptosis and glomerular dysfunction. Another report showed *lncRNA9884* is upregulated in kidneys of diabetic db/db mice, and its expression was positively correlated with DN progression (75). This lncRNA was primarily expressed in glomerular mesangial and tubular epithelial cells and induced in the diabetic milieu *via* Smad3 binding to its promoter. Downregulation of kidney-specific *lncRNA9884* in db/db mice reduced glomerular dysfunction, fibrosis, and proteinuria independent of body weight and blood glucose levels. Mechanistically, nuclear-enriched *lncRNA9884* directly interacts with chromatin at the promoters of inflammatory genes such as *Ccl2* and increases their expression (75). The detailed mechanisms regulating this lncRNA in DN, as well as its human relevance, are not clear.

The role of AGEs and their actions *via* their receptor (RAGE) signaling drives DN pathogenesis. AGEs induced a novel lncRNA *Erbb4-IR* in a Smad3-dependent manner in mouse mesangial cells and tubular epithelial cells. *Erbb4-IR* induced fibrotic genes like collagen *via* sponging the miR-29b (which targets collagens) in mesangial cells and tubular epithelial cells. Expression of *Erbb4-IR* was also increased in db/db mice, and its knockdown *in vivo* ameliorated features of DN, including microalbuminuria, creatinine, and fibrosis (77). LncRNA *MALAT1* was also upregulated (78). Interestingly, nuclear lncRNA *MALAT1* expression was also upregulated in kidneys from DN patients and glomeruli from DN mice. It correlated with reduced levels of *Klotho*, a component of the endocrine fibroblast growth factor (FGF) receptor complex. Reduced levels of *Klotho* are implicated in renal disease (107). A similar inverse relationship between *MALAT1* and *Klotho* was observed in HG-treated human renal glomerular endothelial cells (HRGECs), human renal tubular epithelial cells (HK2), and podocytes (78). Furthermore, *MALAT1* overexpression in HRGECs promoted glucose-induced endothelial cell injury by inhibiting Klotho expression *via* enrichment of G9a and corresponding repressive histone modifications at its promoter.

It is thus becoming increasingly evident that many lncRNAs have critical functions in multiple renal cells, and their dysregulation in diabetes can modulate DN progression. Targeting critical renal lncRNAs with various RNA targeting modalities can confer protection against DN in mice, but more work is needed to extend these approaches for human DN treatment.

## Role of LncRNAs in Diabetic Retinopathy

**Figure 6:**
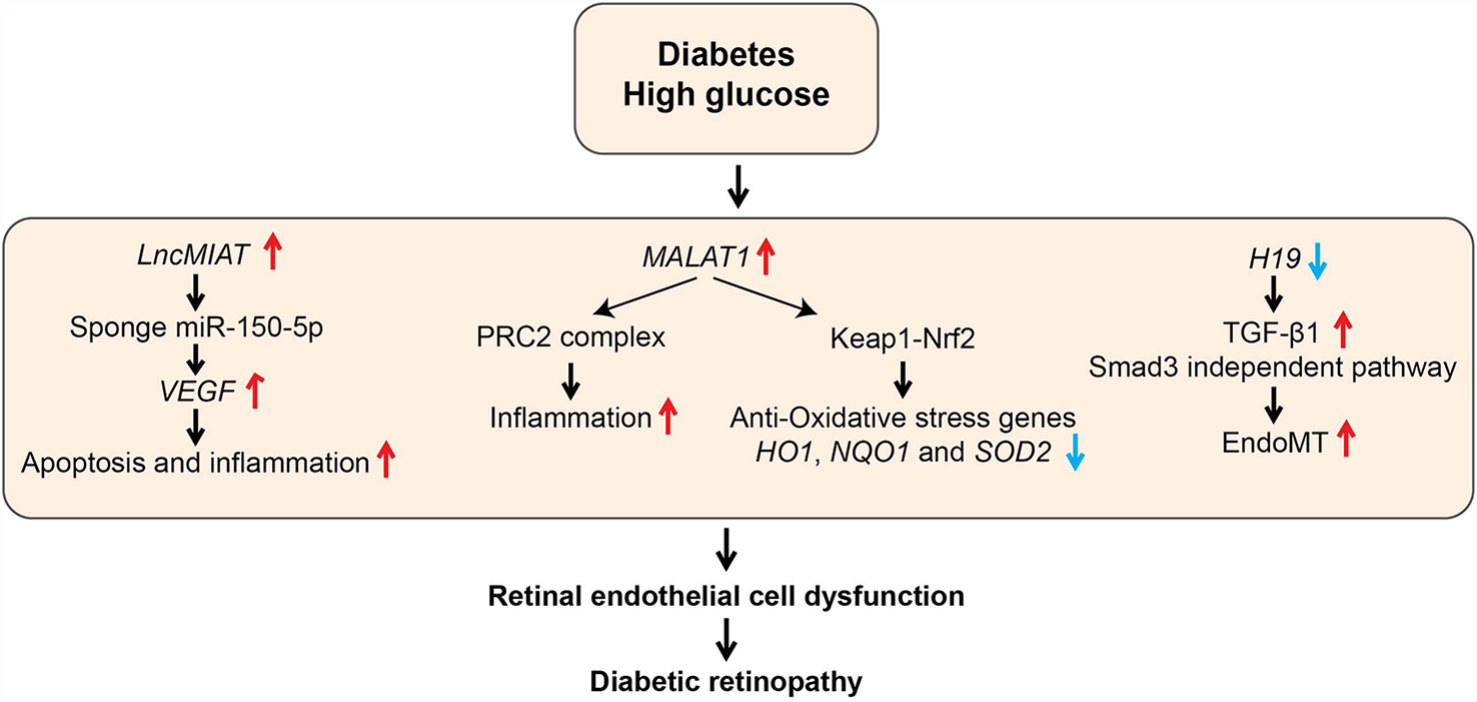
Role of LncRNAs in diabetic retinopathy (DR). The schematic diagram shows the processes involved in the regulation of genes mediated by candidate lncRNAs during various pathological processes associated with DR. EndoMT, Endothelial to mesenchymal transition; HO1, Heme oxygenase-1; Keap1, Kelch Like ECH Associated Protein 1; NQO1, NAD(P)H Quinone Dehydrogenase 1; Nrf2, Nuclear factor erythroid 2-related factor2; PRC2, Polycomb Repressive Complex 2; SOD2, Superoxide dismutase 2; VEGF, Vascular Endothelial Growth Factor.

Diabetic retinopathy (DR) is a major microvascular complication affecting a significant proportion of diabetic individuals and is a major cause of blindness. Dysfunction of retinal ECs and microvascular tissue homeostasis induced by HG, hypoxia, and growth factors are key processes in diabetes-induced retinal damage and retinopathies (108). Several lncRNAs associated with microvascular endothelial dysfunction have been described (**Table 1** and **Figure 6**). Expression of lncRNA *MIAT* was upregulated in retinal tissues from diabetic humans, rat, and mice and in retinal ECs treated with HG (81). Moreover, knockdown of *MIAT* decreased apoptosis, inflammation, and phosphorylation of AKT kinase. Studies in cultured ECs showed that *MIAT* acts as a miR-150-5p sponge and increases the expression of miR-150-5p target, vascular endothelial growth factor (VEGF), a potent angiogenic factor that promotes retinopathy. Further, intraocular injection of siRNAs targeting *MIAT* ameliorated DR in diabetic rats, possibly through *MIAT* knockdown in ECs as well as non-ECs. Since intraocular injections with anti-VEGF antibodies are already in clinical use, in the future, combination therapy with lncRNA-based oligonucleotides could be evaluated for potential superior beneficial effects, especially for patients refractory to anti-VEGF antibodies.

LncRNA *MALAT1* was also demonstrated to have a role in inflammation and retinal EC dysfunction associated with DR. *Malat1* was upregulated in retinas of rats with DR, and its knockdown with short hairpin RNA ameliorated retinal inflammation and retinal vessel impairment in these rats (109). Furthermore, *MALAT1* was also upregulated by HG along with inflammatory cytokines in human retinal ECs (HRECs), which was abolished by *MALAT1* knockdown with GapmeRs (79). In this study, the role of complex epigenetic mechanisms, including *MALAT1* interaction with EZH2 and dysregulation of DNA methyltransferases such as DNMT1 have been implicated in *MALAT1*-dependent and -independent production of HG-induced inflammatory genes in HRECs (79). However, further studies are needed to clarify the precise role of these epigenetic mechanisms in the pathogenesis of human DR. Recent studies showed that *MALAT1* also plays a central role in inhibiting the anti-oxidant defense pathway to promote inflammation by regulating nuclear factor erythroid 2-related factor2 (NRF2), a master regulator of the anti-oxidant defense pathway that protects retinas from oxidative stress. NRF2 function is negatively regulated by Kelch-like ECH-associated protein 1 (KEAP1). But, KEAP1 levels are increased in DR, which enhances oxidative stress and retinal cell death (110). HG upregulated *MALAT1 via* transcriptional activation by Sp1 in HRECs. In parallel, HG also increased KEAP1 and inhibited nuclear translocation of Nrf2 as well as transcription of anti-oxidant genes *HO1* and *SOD2*. *MALAT1* knockdown with siRNAs abrogated all these HG-induced effects in HRECs (80). Furthermore, similar changes in *MALAT1*, *KEAP1*, and anti-oxidant genes were also demonstrated in retinal microvessels from diabetic rats and humans with DR (80). These studies collectively support the notion that *MALAT1* promotes inflammation and oxidative stress in DR, and *MALAT1* could be a viable target for DR treatment.

Endothelial to mesenchymal transition (EndoMT) also plays an essential role in EC dysfunction and development of DR. EndoMT can be induced by TGF-β signaling, oxidative stress, and hypoxia, and leads to loss of EC phenotype and acquisition of mesenchymal cell features, including increased inflammatory cytokines, proliferation, and migration. Accumulating evidence suggests a role of lncRNAs like *H19* in EndoMT in the pathogenesis of DR. *H19* was downregulated in retinas of humans with DR and *H19* knockout could exacerbate DR in mice. Mechanistic studies in retinal ECs showed that downregulation of *H19* by HG promotes TGF-β1 -induced EndoMT. In contrast, *H19* overexpression inhibited EndoMT *via* downregulation of TGF-β1 expression (82). These results suggest protective functions for *H19* in DR. Dysregulation of lncRNAs *MEG3* (Maternally Expressed Gene 3) and *ANRIL* (Antisense Non-coding RNA in the INK4 Locus) in retinal cells also contributed to increased inflammation in DR. However, detailed mechanisms of action of these lncRNAs were not examined (83–85). These reports, taken together, illustrate the involvement of lncRNAs in controlling retinal microvascular EC homeostasis and pathology in DN.

## Approaches to Target LncRNAs *In Vivo*

As detailed in the earlier sections, it is clear that dysregulated expression and functions of multiple lncRNAs play pivotal roles in the pathogenesis of various diabetic vascular complications. Thus, developing therapies targeting lncRNAs and their key effector molecules can have tremendous translational potential. Recently, RNA-based therapies have been gaining a lot of momentum in general, but relatively much less progress has been made in the field of diabetes complications. Unlike miRNAs that can be targeted quite easily *in vitro* and *in vivo*, targeting lncRNAs can be more challenging, especially *in vivo*, due to their relatively large size, complex structure, low expression, tissue/cell specificity, and low conservation across species. The expression of lncRNAs can be modulated using RNA interference (RNAi) with siRNAs, small antisense oligos (ASOs), or CRISPR-Cas9 based genome editing of lncRNA loci. Endogenous lncRNA expression can be enhanced using small activating RNAs (saRNAs) or by guiding transcriptional activators to lncRNA gene promoters using CRISPR-Cas9 genome editing (111–113).

RNAi approaches utilize siRNAs, which are 20–25 nucleotides long double-stranded ribonucleotides that, after delivery into the cytoplasm, are loaded into the RNA-induced silencing complex to induce target RNA degradation. ASO approaches mostly use LNA-modified GapmeRs, 16-nucleotide long single-stranded oligos that are DNA-RNA hybrids containing a central DNA sequence (Gap) flanked by LNA nucleotides (72, 114, 115). The central DNA hybridizes with the complementary sequence in the target lncRNA and initiates the cleavage of target lncRNA by RNase H-dependent mechanisms (114). LNA modifications increase affinity and base pairing specificity of the GapmeRs, while phosphorothioate backbones confer resistance to degradation by endogenous nucleases (114). In contrast, saRNAs are used to overexpress endogenous lncRNAs or to restore normal levels when they are downregulated under different disease states. saRNAs are 21-nucleotide long double-stranded RNAs with two nucleotide overhangs and contain complementary sequences to the promoter regions. After delivery into cells, they are loaded into the saRNA-AGO2 complex, which enters the nucleus and binds to the target promoter and activates transcription (113). Each of these methods has its own merits and pitfalls, and lncRNA knockdown efficiency is also influenced by subcellular localization. siRNAs are, in general, very efficient, and design algorithms have been well developed, but they may not be as efficient as GapmeRs to knockdown nuclear lncRNAs. On the other hand, LNA GapmeRs working *via* RNase H mechanisms can efficiently target both nuclear and cytoplasmic lncRNAs. In addition, dual localized lncRNAs may require both siRNAs and GapmeRs for efficient knockdown (115). However, GapmeR design algorithms are less well developed compared to siRNA design; therefore, they require testing of several GapmeRs before an optimal one can be selected. In addition, a recent study showed that targeting lncRNAs with GapmeRs at the 5’ sequence could lead to immature transcriptional termination (116). In addition, dual localized lncRNAs may require both siRNAs and GapmeRs for efficient knockdown. Recently, siRNA-based therapies have reached clinical development; the chemically modified siRNA targeting PCSK9 (Inclisiran) for CVD showed an efficient reduction in LDL levels in Phase II clinical trials (117). As described earlier in this review, GapmeRs targeting lncRNAs have been used in animal models of diabetes and in human cells to ameliorate features of diabetic complications (72, 118, 119), thus, providing proof of principle for future evaluation in human clinical trials. However, extensive studies are needed to determine safe and efficient delivery vehicles and organ-, tissue-, or cell-specific accumulation of GapmeRs to reduce off-target effects before they are evaluated in clinical trials.

CRISPR-Cas9 editing is increasingly emerging as a viable approach to address some concerns with RNA therapies while also providing potentially long-term gene editing at specific loci. CRISPR-Cas9 editing can be used for a partial or complete deletion of the lncRNA locus. Locus-specific CRISPR activation (CRISPRa) or interference (CRISPRi) methods are also widely used to overexpress or inhibit lncRNA expression, respectively. In these approaches, catalytically dead Cas9 nuclease is fused with transcriptional activator or repressor proteins, which are directed to specific lncRNA loci by guide RNA (gRNA)s (111, 120). The CRISPRa or CRISPRi modifies the chromatin environment at the promoters, leading to activation or repression of lncRNAs transcription. However, CRISPR-Cas9 editing also has its own limitations (121), including off-target effects, but recent technical advances have significantly improved specificity (121). Other challenges include difficulty in achieving cell-type-specific targeting and the possibility of unanticipated side effects due to long-term alteration of lncRNA expression. Furthermore, unlike coding genes, deleting the first exon of a lncRNA gene may not silence specific lncRNA completely, and alternate transcripts with similar activity may still be expressed. Since lncRNAs with multiple exons may occupy large genomic regions, and deletion of the entire locus may elicit unintended deleterious effects. Additionally, targeting bidirectional and lncRNAs overlapping with the promoter of a neighboring coding gene *via* CRISPR-Cas9 editing may inadvertently deregulate neighboring genes leading to undesirable effects. For such lncRNAs, RNAi or ASO-mediated knockdown approach would be more appropriate (122). However, there has been significant recent progress in the direct clinical application of CRISPR-Cas9 editing (123). These reports offer great promise for CRISPR-based therapy to correct defective coding or ncRNAs for human diseases, including diabetic complications.

Of note, modulation of lncRNA expression can also be achieved *via* transfecting lncRNA transcripts into cells or *via* injecting viral vectors into the mice. Recombinant adeno-associated virus (rAAV)-based gene therapy is now gaining interest and in various stages of clinical trials for the treatment of multiple diseases (124). The viral vector transgenes up to ~5 kb flanked by AAV inverted terminal repeats are inserted into rAAV genomes (125). The hepatic delivery of lncRNA *Lexis* in mice models of hypercholesterolemia and atherosclerosis to overexpress *Lexis* led to a significant reduction of hypercholesterolemia and ameliorated atherosclerosis burden versus control vector treated mice (126). These reports demonstrate the feasibility of lncRNA mimetic therapeutic strategy for diabetic CVDs, such as atherosclerosis and possibly other diabetes complications. One advantage of rAAVs is that, unlike adenoviral vectors, they are less immunogenic and widely used in *in vivo* settings (124). Most of these methods have been applied for targeting the mRNA or DNA sequence of a single gene implicated in monogenic diseases. However, extending them for the treatment of polygenic, multifactorial diseases and complex disorders, like diabetes and its complications, requires further investigation of precise cell-specific targeting approaches, optimal design for enhanced bioavailability, reduced toxicity, and efficient delivery into target tissues.

## Summary and Perspectives

LncRNAs are emerging as not only important regulatory RNAs that function in many cellular processes during normal states but also as critical modulators of disease states. They can play significant roles in the pathology of diabetic complications by regulating genes that affect inflammation, fibrosis, ER stress, oxidative stress, and mitochondrial dysfunction. With rapid advances in sequencing technologies, transcriptomics, and epigenomics, as well as bioinformatic tools, we will undoubtedly continue to identify more lncRNAs involved in the development of diabetic complications. Advances in single-cell sequencing will be invaluable to identify lncRNAs expressed in distinct cell types in diabetes, which, in turn, would enhance the development of targeted therapies. Interestingly, data analysis of 10 transcriptomes from granulocytes from different individuals revealed that lncRNAs expression varies amongst individuals at levels that are greater than protein-coding genes (127). It will be useful to understand whether expression of a specific lncRNA is associated with an individual’s susceptibility to diabetic complications. Additionally, valuable catalogs of lncRNAs expressed in normal cells and tissues are available (128). These data sets can be compared to datasets from transcriptomic analyses of cells and tissues from diabetic patients to uncover dysregulated lncRNAs to gain more insights into their function in diabetic vascular complications.

In addition to dysregulated lncRNAs, genetic variation within lncRNA regions can modulate function of lncRNAs and drive disease pathogenesis. Most of the genetic variants associated with cardiometabolic traits and diabetes are present in non-coding regions of the genome. Several hundreds of lncRNA regions harbor disease-associated single nucleotide polymorphisms (SNPs), suggesting the importance of these lncRNA regions in disease traits (129). A recent study mapped regulatory variants that modulate DNA binding of the coronary artery disease-associated TF TCF21 (130). Integration of epigenome-wide association studies (EWAS) and GWAS identified several genetic variations that correlated with epigenetic mechanisms associated with TCF21 binding in human coronary artery VSMC (130, 131). Because, TCF21 function is also dysregulated in diabetes complications, these and other reports suggest a link between genetic and epigenetic variations in modulating vascular complications (131, 132). Similar studies with SNPs in lncRNA loci and cis-regulatory elements regulated by them can further advance our understanding of the regulation and functional roles of lncRNAs in diabetes complications. Studies from our group showed that enhancers, super-enhancers, and eRNAs are dysregulated in VSMCs treated with AngII (133). These enhancers harbored SNPs associated with CVDs. Since lncRNAs can regulate the functions of these enhancers and super-enhancers, any genetic variations affecting enhancer activity can alter lncRNA functions. Such chromatin-lncRNA cross-talk and epigenetic mechanisms could be involved in vascular cell dysfunction associated with diabetic CVD. Moreover, although eRNAs are implicated as important regulators of gene expression, their function in diabetes complications is not fully clear. Recent studies showed that enhancer associated lncRNAs such as *CARMN* and *LEENE* regulate cardiac cell differentiation and EC function respectively (57, 134). It likely that additional eRNAs will be characterized under diabetic conditions in the upcoming years.

One impediment in extending lncRNA-based studies to the clinic is that lncRNAs are either not expressed in all species or exhibit low sequence conservation across species. For example, *lncADAL* and *CHROME*, which regulate adipocyte differentiation and cholesterol homeostasis, respectively, are primarily expressed only in humans. Whereas *lnc-MGC* is expressed in both mice and humans and have similar functional roles related to DN, but their nucleotide sequences are not highly conserved (53, 72, 135). However, evidence shows that, even in the absence of nucleotide sequence conservation, secondary structures and lncRNA functions can be conserved across species (136). Interestingly, evidence shows non-conserved lncRNAs harbor a significant number of SNPs associated with cardiometabolic traits than conserved lncRNAs, which can enhance our understanding of the roles of key SNPs and lncRNAs in driving disease pathogenesis (129). Moreover, lncRNAs expressed from syntenic gene loci often showed limited sequence conservation but had functional similarities in mice and humans (27, 29, 49, 52). These studies demonstrate the importance of prioritizing not only conserved but also non-conserved lncRNAs for future studies. Notably, a recent study demonstrated that the function of non-conserved human lncRNAs in metabolism can be studied *in vivo* using a humanized liver mouse model (137). Therefore, investigations are likely to also focus on lncRNA functions and functional lncRNA domains conserved across species through prediction of secondary structure using bioinformatics tools and experimental approaches, such as humanized mice and domain-specific chromatin isolation by RNA purification (dChIRP) (136–138). Identification of lncRNAs harboring genetic variations associated with diabetes complications could provide important insights into the cross-talk between transcriptomics, genetics, and epigenetics underlying diabetic complications that could inform the development of much needed novel therapeutic strategies. Additionally, lncRNA expression profiles in circulating biofluids can yield valuable non-invasive biomarkers for early detection of diabetic complications, a major unmet need in clinical management. With the rapid advances in genomic sequencing, single-cell sequencing, spatial transcriptomics, and RNA targeting approaches, lncRNA research is clearly expected to grow exponentially in the upcoming years.

## Author Contributions

VT, MR, and RN conceived and designed the study. VT, MR, and RN wrote and critically revised the manuscript for important intellectual content. VT prepared the figures. All authors contributed to the article and approved the submitted version.

## Funding

We gratefully acknowledge funding from the National Institutes of Health (NIDDK and NHLBI) R01 DK065073, R01 HL106089, R01 DK081705, DP3 DK106917, the Schaeffer Foundation, and the Wanek Family Project for the Cure of Type 1 Diabetes at City of Hope.

## Conflict of Interest

The authors declare that the research was conducted in the absence of any commercial or financial relationships that could be construed as a potential conflict of interest.
